# Formative evaluation of a training intervention for community health workers in South Africa: A before and after study

**DOI:** 10.1371/journal.pone.0202817

**Published:** 2018-09-24

**Authors:** Alexandra Plowright, Celia Taylor, David Davies, Jo Sartori, Gillian Lewando Hundt, Richard J. Lilford

**Affiliations:** 1 Warwick Medical School, University of Warwick, Coventry, United Kingdom; 2 Educational Development & Research Team, University of Warwick, Coventry, United Kingdom; Boston University School of Public Health, UNITED STATES

## Abstract

**Background:**

Community Health Workers (CHWs) have a crucial role in improving health in their communities and their role is being expanded in many parts of the world. However, the effectiveness of CHWs is limited by poor training and the education of CHWs has received little scientific attention.

**Methods:**

Our study was carried out in two districts of KwaZulu-Natal, South Africa. We developed and piloted an inexpensive (two day) training intervention covering national government priorities: HIV/AIDS, sexually transmitted disease and Tuberculosis; and Women’s Sexual and Reproductive Health and Rights. Sixty-four CHWs consented to participate in the main study which measured knowledge gains using a modified Solomon design of four different testing schedules to distinguish between the effects of the intervention, testing and any interaction between intervention and testing. We also measured confidence, satisfaction and costs.

**Results:**

Following the training intervention, improvements in knowledge scores were seen across topics and across districts. These changes in knowledge were statistically significant (p<0.001) and of large magnitude (over 45 percentage points or four standard deviations). However, the CHWs assigned to the test-test-train schedule in one district showed high gains in knowledge prior to receiving the training. All CHWs reported high levels of satisfaction with the training and marked improvements in their confidence in advising clients. The training cost around US$48 per CHW per day and has the potential to be cost-effective if the large gains in knowledge are translated into improved field-based performance and thus health outcomes.

**Conclusion:**

Training CHWs can result in large improvements in knowledge with a short intervention. However, improvements seen in other studies could be due to test ‘reactivity’. Further work is needed to measure the generalisability of our results, retention of knowledge and the extent to which improved knowledge is translated into improved practice.

## Introduction

Community Health Workers (CHWs) have played a major role in improving health in many low- and middle-income countries (LMICs).[1] In Sub-Saharan Africa, CHWs have made a significant contribution to the fight against HIV/AIDS and tuberculosis by linking and retaining patients in appropriate care and helping them adhere to long-term therapy.[2] As the HIV epidemic comes under control, and inspired by the wider contributions that CHWs have made to healthcare in Asia and South America,[3] African countries are increasingly promulgating policies to employ more CHWs and increase their range of tasks.[4,5] Critical to the success of CHW programmes is the provision of high-quality initial and ongoing training.[6] However, some CHWs do not receive a sufficient quantity of quality of training.[7,8] As a result, their knowledge has often been found to be inadequate when assessed.[9,10]

In this paper we report on the development and evaluation of a scalable educational intervention to reinforce and extend the knowledge of CHWs employed in KwaZulu-Natal, South Africa. South Africa’s health guidelines prioritise certain topics for the ongoing training of CHWs. We selected two topic groups from these guidelines: HIV/AIDS, Sexually transmitted disease and Tuberculosis (HAST), and Women’s Sexual and Reproductive Health and Rights (WSRHR). The principle aims of our project were to:

1. Determine whether, and to what extent, a short (two day) training course designed to meet local needs could improve knowledge among CHWs.
2. Examine the extent to which testing was itself an educational tool (i.e. examine test ‘reactivity’).
3. Determine the extent to which CHWs’ knowledge would improve over a module covering topics with which they were familiar from the workplace (HAST) vs. a module containing a higher proportion of new material (WSRHR).
4. Estimate the relationship between individual CHWs’ baseline knowledge and the extent to which new knowledge was acquired.
5. Measure the change in confidence following education, CHWs’ satisfaction with the education provided, and estimate the relationship between post-intervention knowledge and confidence.
6. Determine the cost of running the course.

## Materials and methods

The protocol for this study can be found at: http://www.warwick.ac.uk/fac/med/about/centres/cahrd/lay_community_health_workers/

### Study setting

The setting for the project was two districts (A and B) in KwaZulu-Natal province in South Africa. KwaZulu-Natal has a well-developed CHW system. These districts were selected because the project partner, Sizabantu, a South African registered non-governmental organisation (NGO), had a strong presence in the districts, already operated a home-based care service provided by CHWs, and provided some training to NGO employed or associated CHWs. Box 1 has a description of each district.

**Box 1: Description of the two districts**

District A is located in the interior of KwaZulu-Natal. Each community area has a male-led committee, which focusses on advocacy for the provision of, and access to, various services, including healthcare, mostly associated with HIV/AIDS. The committees operate in collaboration with the Department for Cooperative Governance and Traditional Affairs (CoGTA) and the relevant local municipality. The district is served by a government health clinic based outside the district, and a mobile clinic, headed by a staff nurse, visits the district every six weeks. CHWs are supported by Sizabantu, other NGOs, or the KwaZulu-Natal Department of Health. However, there are no ward-based outreach teams operating in this district.

District B is located on the coast of KwaZulu-Natal. The district is characterised by high migration, both domestic and regional. People move as individuals and families come to the area in search of employment opportunities on sugar cane farms and in tourism. Resources and infrastructure are provided by the government, and coordinated by a representative from CoGTA and associated committees. The diversity of the population in the district and area-level management results in a disparity in service provision between areas. CHWs linked to Sizabantu, other NGOs, and some who are government-employed operate across four community areas. Again, there are no ward-based outreach teams in operation.

In both districts, Department of Health-employed CHWs are linked to primary healthcare clinics but the allocation of CHWs to households is done informally.

### The intervention and its development

Intervention design conformed with established principles, for example the recent WHO and United Nations Population Fund Report.[11] The intervention was designed to align to national health priorities and the needs of the CHWs, which were explored qualitatively in the first phase of the project. CHWs were involved in design of the training, which was adapted to local needs. Intervention development proceeded through three stages: curriculum design, design of an intervention to deliver the curriculum, and pilot implementation.

#### 1. Curriculum

The curriculum encompassed two topics (HAST and WSRHR) each covering a focus area designated by the National Government, so the (top-down) starting point was a review of the existing KwaZulu Natal CHW training manual. This existing curriculum was subsequently developed by triangulation with consultation with domain experts, Department of Health professional staff, and CHWs. In this way we sought to ensure that the curriculum covered material necessary to understand each topic and would help CHWs meet the needs of their communities as reported by health officials, supervisors and clinic managers, conformed with national policy requirements, and met the expressed (bottom-up) needs of the local CHWs themselves (i.e. their expressed opinions of what they felt was missing from their training and what and how they would prefer to learn).

#### 2. Design of an intervention to deliver the curriculum

This stage consisted of two tasks. First, teaching materials were developed to represent the curriculum. Again, advice was sought from Department of Health employees and domain experts. These materials are available online at: http://www2.warwick.ac.uk/fac/med/about/global/etatmba/training/, under a Creative Commons license. Second, a training structure was devised in consultation with CHWs.

#### 3. Pilot intervention

The pilot consisted of two workshops, one for each module, each delivered over two days with about five hours of contact and testing time each day. The intervention was delivered in the local language, isiZulu, by AP who was supported by topic area experts and two translators. The intervention was evaluated using multiple methods–multiple choice (single best answer) tests before and after each training session, CHWs’ ratings of their confidence to advise clients and their perceptions of the topics, semi-structured interviews with each participant CHW, and focus groups [total N = 16]. A number of changes were made as a result. First, we increased participant engagement, so that the teaching style became more interactive and less didactic. Second, we adapted delivery according to different learning needs across the districts. For instance, the topic of intimate partner violence needed to be approached with particular sensitivity in the more traditional district A. Third, we found that participants struggled with multiple choice tests, so we opted for a ‘yes, no, don’t know’ format in the subsequent study. Fourth, to keep costs to a minimum we increased the size of each training group from eight in the pilot to 16 in the main study, which we now describe.

### Participants

The CHWs were selected for participation in the main study through an advertisement in a local newspaper in each district. CHWs from any employment background (inclusive of NGO and government employed CHWs) were invited to apply to participate and we received 91 responses. The first 32 CHWs who responded from each district (and whose CHW programme providers agreed that their CHWs could participate) were selected, giving a total of 64 participants. Daily transport to and from the training venues and refreshments were provided. No financial incentive was offered nor were fees charged.

Each of the 64 participating CHWs were given a unique identifier number for their participation in the study. Within each district, the 32 participants were divided into two groups of 16 based on location. Each participant was invited to attend two workshops covering the HAST and WSRHR topics, each over two days. In district A the workshops were held in a community hall and a church hall and in district B the workshops were held in a community hall. These venues were chosen because they were large enough to accommodate all participants, had electricity and were affordable.

### Data collection: Test scores, self-rated confidence and satisfaction with the course

Knowledge tests were devised for each topic [see S1 File]. The same test was used before and after the intervention. Every correct answer was given a score of +1, every incorrect answer -1, and every ‘don’t know’ answer 0. The maximum possible score was 56.

Confidence in advising clients on topics covered in the training was elicited by means of a questionnaire both before and after each of the training sessions [see S2 File]. Each question was rated on a five point Likert-type scale, with 1 indicating the lowest level of confidence and 5 the highest. An aggregate rating across the three questions for each workshop was generated for each time point; this rating could range from 3 (low) to 15 (high).

Each participant was also asked to complete a satisfaction questionnaire of five questions [see S3 File] following each module, again rating each question on a five point Likert-type scale.

The test score dataset analysed during this study is available from Mendeley (https://data.mendeley.com/datasets/2zr7k8w5vc/1).

### Allocation of participants to pre-defined testing and intervention sequences

**Fig 1:**
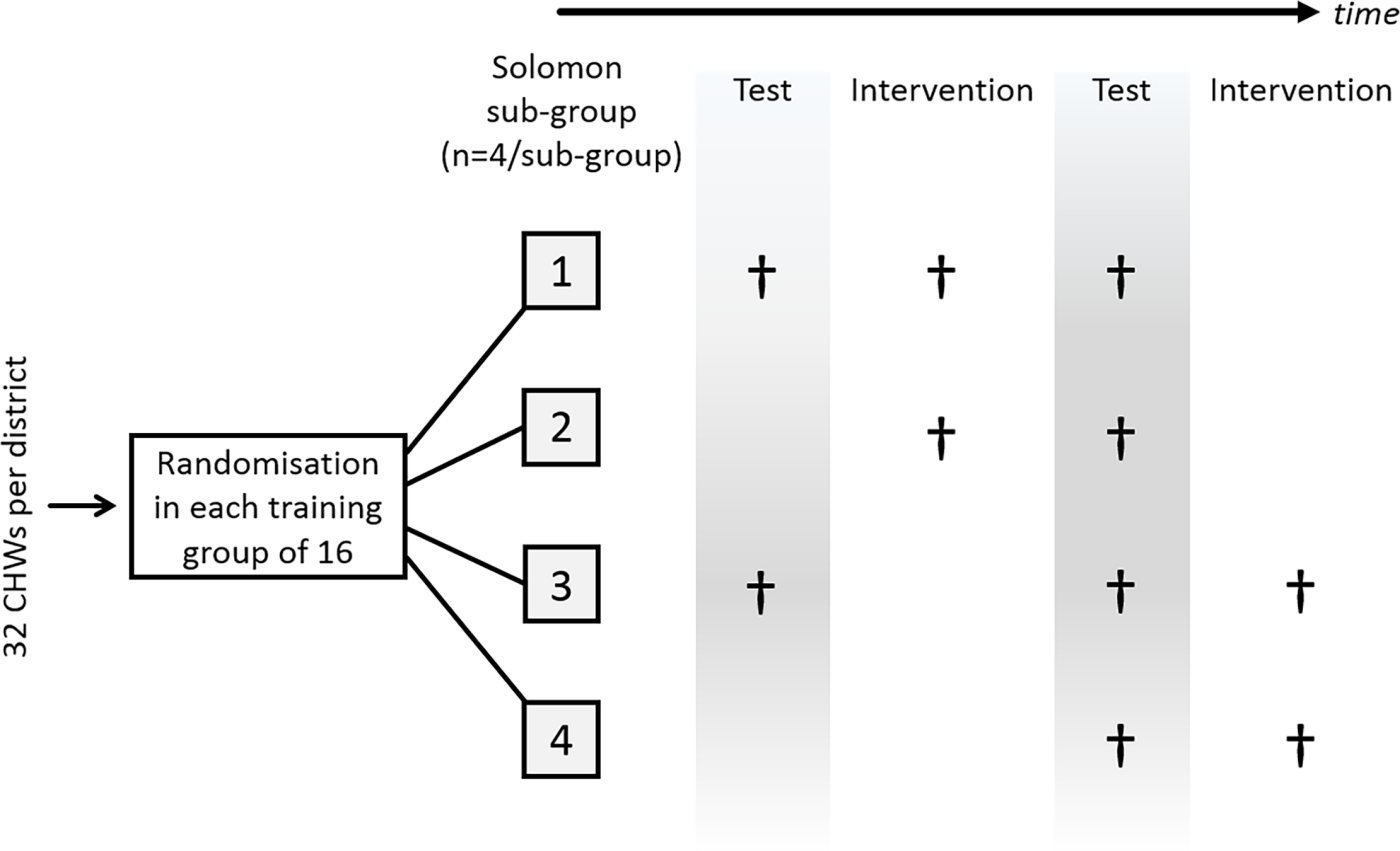
Representation of the modified Solomon design. In the original Solomon design,[13] groups 3 and 4 would not receive the intervention following the second test interval.

We used a modified Solomon four-group design to mitigate the bias introduced by interaction between taking part in a pre-test and the training–the problem of ‘reactivity’.[12] The modified Solomon Design is shown in Fig 1. The modification to the ‘pure’ Solomon design ensures that no CHW was excluded from receiving the intervention. Four CHWs in each group of 16 CHWs were randomised to each of the four ‘Solomon sub-groups’ by picking names from a hat. We considered this informal method of randomisation suitable for a field experiment. The names were shuffled in public view and pre-test measurements were compared as a ‘test’ of this procedure.

### Data collection: Costs

We designed a resource use and cost data pro-forma including all the individual resources required to deliver the workshop.[14] Actual costs were given in South African Rand (2016) using ‘shadow’ costs where required (e.g. to hire a computer when in reality the researcher already had one). Calculations were made including and excluding CHW time costs. We did not include the cost of developing the workshops through the three preliminary phases described above.

### Analysis plan

Table 1 summarises the analysis plan.

**Table 1:** Analysis plan.

Study aim	Data used	Solomon sub-groups included	By module or combined	Districts aggregated or separated	Method(s) of analysis (using Stata v14)
1. Evaluate improvement in knowledge	Knowledge test scores*	1,3	By module	Separated	Effect size by Solomon sub-group. Independent samples t-test of change in score by Solomon sub-group (differences in differences).
2. Examine test ‘reactivity’	Knowledge test scores (post)	1,2,3,4	By module	Separated	2x2 between-groups analysis of variance on post-test scores with factors receipt of training (prior to test), sitting of pre-test and their interaction.[15] A statistically significant interaction term suggests ‘reactivity’ bias.
3. Compare knowledge gains for HAST (previous experience) and WSRHR (new)	Knowledge test scores	1	N/A	Aggregated	Paired t-test of change in score for each module.
4. Estimate relationship between pre-test knowledge scores and knowledge gain	Knowledge test scores	1	By module	Aggregated	Pearson’s correlation coefficient.
5.A) Measure change in confidence	Confidence ratings* (aggregated across questions)	1,2,3,4	By module	Separated	Wilcoxon signed-rank test of pre and post scores.
5. B) Evaluate CHWs’ satisfaction with the course	Satisfaction ratings (post)	1,2,3,4	By module	Separated	Percentage of CHWs reporting that the workshop was ‘definitely’ or ‘quite’ satisfactory for each of the five satisfaction rating questions.
5. C) Estimate relationship between knowledge and confidence	Knowledge test scores (post) Confidence ratings, aggregated across questions (post)	Post: 1,2	By module	Separated	Kendall’s tau-b correlation coefficients.
6. Evaluate cost	Costs data	1,2,3,4	Combined	Aggregated	See below.
Additional analyses					
Assessment of randomisation	Knowledge test scores (pre)	1,3,4	By module	Separated	Visual comparison of means and standard deviations.
Comparison of pre-test knowledge scores across districts	Knowledge test scores (pre)	1,3,4	By module	N/A	Independent samples t-test of pre-test scores by district.
Internal consistency and standard error of measurement	Knowledge test scores (post)	1,3	By module	Aggregated	Cronbach’s alpha (0 = no reliability; 1 = perfect reliability).
Intra-cluster correlation	Knowledge test scores (pre and post)	Pre: 1,3,4 Post: 1,2	By module	Aggregated	Intra-cluster correlation computation.

* Knowledge test scores were approximately normally distributed so parametric testing was employed. Aggregated confidence ratings were positively skewed at post-test so non-parametric testing was employed. Analyses combined both pre and post training data unless otherwise specified.

Statistical analysis was undertaken using Stata v14. Because of the number of statistical tests being undertaken, a p-value of <0.005 was used to determine statistical significance using two-tailed tests. The study was designed as an “Intervention development” project, rather than a hypothesis testing project and no sample size calculation was undertaken. However, we thought it important to provide a measure of the precision and statistical significance of the parameter estimates.

### Analysis of cost of training

The value of CHWs’ time was estimated based on a stipend of US$60 per month for a CHW working 3.5 days per week. All costs collected in South African Rand were converted to US$ using Purchasing Power Parity and local currency unit exchange rates.[16] Total costs, cost per CHW and costs per day of training per CHW were then calculated, both including and excluding CHW time costs.

### Ethics approval

Ethical approval for the project was sought from two institutions, the University of Warwick Biomedical and Scientific Research Ethics Committee and locally from the South African Human Sciences Research Council Research Ethics Committee (HSRC-REC). The Warwick ethics approval was provided for the programme duration, with the project registration code REGO-2015-1663. The ethical clearance code for the obtained approval from HSRC-REC is 1/8/11/15, which was valid until 17 February 2018, by which time the study was completed. The unique identifier number used for each CHW ensured that data were anonymous. All CHWs provided written informed consent to participate in this study and none were aged under 18. Approval to conduct this study in South Africa was obtained from the South African Human Sciences Research Council. There was no need for additional permission to undertake this research outside of that provided by the South African Health Sciences Research Council because we worked through the South African organisation Sizabantu. AP did not count as a ‘foreign researcher’ in terms of South African law owing to her status as trustee of Sizabantu.

## Results

### Participants

All 64 participating CHWs took part in all testing/training sessions–there were no drop-outs. Characteristics of participants are given by district in Table 2. The gender balance was similar across districts and just under half were male. CHWs covered a broad age range in both districts, but were slightly younger on average in district B. Twenty five (39%) of the CHWs were employed by an NGO and 39 (61%) employed or associated with the Department of Health. CHWs were reluctant to disclose their prior educational attainment for fear that this may affect their ongoing employment. However, our qualitative data suggested that educational levels were generally lower in district B than district A.

**Table 2:** Participant characteristics.

	District A	District B
N	32	32
**Male**	15 (46.9%)	14 (43.8%)
**Age Group**		
18–29	6 (18.8%)	4 (12.5%)
30–44	8 (25.0%)	19 (59.4%)
45–54	9 (28.1%)	7 (21.9%)
55+	9 (28.1%)	2 (6.3%)
**Rewards**		
Salary	3 (9.4%)	5 (15.6%)
Stipend	13 (40.6%)	17 (53.1%)
Unpaid	16 (50.0%)	10 (31.3%)
**Employment**		
Government	17 (53.1%)	22 (68.8%)
NGO	15 (46.9%)	10 (31.3%)

There were no consistent differences in pre-test scores between CHWs in each Solomon sub-group taking the pre-test (Table 3). In district A, CHWs in Solomon sub-group 1 had lower scores for HAST than those in sub-groups 3 and 4; while those in sub-group 4 had lower scores for WSRHR than CHWs in sub-groups 1 and 3. In district B, CHWs in Solomon sub-group 3 had lower scores for HAST than those in sub-groups 1 and 4.

**Table 3:** Mean (SD) test scores (/56) by district, workshop, Solomon sub-group and time.

	District A						District B					
		Test	Intervention	Test	Intervention	Change		Test	Intervention	Test	Intervention	Change
HAST	Solomon sub-group 1	11.25 (7.50)	x	38.50 (5.45)		27.25 (3.65) ES = 4.16, t = 21.1 p<0.001	Solomon sub-group 1	9.75 (6.25)	x	35.38 (6.05)		25.63 (5.07) ES = 4.17, t = 14.3 p<0.001
	Solomon sub-group 2		x	42.63 (4.10)			Solomon sub-group 2		x	36.75 (3.41)		
	Solomon sub-group 3	19.13 (6.79)		16.88 (8.10)	x	-2.25 (5.47) ES = 0.30, t = 1.16 p = 0.283	Solomon sub-group 3	8.13 (8.31)		36.88 (7.95)	x	28.75 (9.19) ES = 3.53, t = 8.85 p<0.001
	Solomon sub-group 4	19.98 (5.96)			x		Solomon sub-group 4	9.50 (9.90)			x	
WSRHR	Solomon sub-group 1	19.13 (6.24)	x	40.88 (4.02)		21.75 (3.62) ES = 4.14, t = 17.0 p<0.001	Solomon sub-group 1	8.75 (6.48)	x	32.75 (5.42)		24.00 (5.87) ES = 4.02, t = 11.6 p<0.001
	Solomon sub-group 2		x	37.38 (5.50)			Solomon sub-group 2		x	35.13 (5.06)		
	Solomon sub-group 3	17.00 (8.38)		12.00 (5.76)	x	-5.00 (7.25) ES = 0.70, t = 1.95 p = 0.092	Solomon sub-group 3	13.00 (9.37)		34.50 (8.86)	x	21.5 0 (11.44) ES = 2.36, t = 5.32 p<0.001
	Solomon sub-group 4	12.38 (5.83)			x		Solomon sub-group 4	12.50 (8.42)			x	

ES: Effect size (using pooled standard deviation)

CHWs in district A had statistically significantly higher pre-test scores than CHWs in district B for HAST (difference in means 7.6/56, t = 3.39, p = 0.001) and higher scores, but not statistically significantly so, for WSRHR (difference in means 4.8/56, t = 2.15, p = 0.037).

### Test scores, intervention effectiveness and reactivity bias

The internal consistency of post-test scores amongst Solomon sub-groups 1 and 2 was low (Cronbach alphas of 0.29 for HAST; 0.35 for WSRHR). Combined with standard deviations of test scores of around five marks (out of 56), this implies a standard error of measurement of approximately four marks (seven percentage points) and gains lower than this level could be the result of a lack of reliability of the test (i.e. measurement error).

As shown in Table 3, the mean improvement in knowledge scores for both topics and in both districts for CHWs in Solomon sub-group 1 was around 25 marks (45 percentage points; effect sizes of at least four standard deviations) and considerably greater than the standard error of measurement. While there was no evidence of ‘reactivity’ of the test in district A (the interactions between pre-test and intervention were not statistically significant: F = 0.07, p = 0.795 for HAST; F = 1.06, p = 0.312 for WSRHR), there was a large effect of the test alone in district B (interactions: F = 31.6, p<0.001 for HAST; F = 23.3, p<0.001 for WSRHR).

In district A, CHWs in Solomon sub-group 1 improved statistically significantly more than those in sub-group 3 (results of t-tests: HAST t = 12.68, p<0.001; WSRHR t = 9.34, p<0.001, with absolute differences in differences in test scores of around 28 marks or 50 percentage points). In district B, CHWs in Solomon sub-group 3 also showed large improvements in scores despite not receiving the intervention until after the second test, so there was no difference in knowledge gains between the two sub-groups (results of t-tests: HAST t = -0.84, p = 0.415; WSRHR t = 0.55, p = 0.591).

Topics which appeared to be hard to assimilate (from low post-test scores) were appropriate pathways for people needing referral (HAST) and cervical smears (WSRHR). Improvements in test scores for CHWs in Solomon sub-group 1 were slightly higher for HAST than for WSRHR, despite WSRHR being a relatively new topic, although the difference was not statistically significant (paired t-test, combining districts: t = 2.19, p = 0.037).

The intra-cluster correlations were: HAST pre-test 0.30, post-test 0.26; WSRHR pre-test 0.13, post-test 0.31.

### Relationship between pre-test scores and improvements in scores

There was a negative relationship between pre-test scores and improvements following the intervention for both topics, which was stronger for WSRHR than for HAST (r = -0.52 p = 0.038 for HAST; r = -0.65, p = 0.007 for WSRHR, both N = 16). This suggests that the workshop content was accessible to all CHWs regardless of pre-test knowledge scores. However, it also suggests that the content may have been insufficiently challenging for those with higher pre-test knowledge scores, although the headroom for improvement also falls as pre-test scores increase.

### Confidence ratings, satisfaction with training and correlation between confidence rating and knowledge test score

The positive impact of each training workshop on the aggregate confidence ratings in each district is shown in Table 4. The difference between topics (greater increase for WSRHR than for HAST) would be expected as CHWs tended to have previously been trained on HIV and TB (part of HAST) and had identified a particular training need for the WSRHR topic. The improvement in confidence applied more or less equally across the three questions in each topic area (data not shown).

**Table 4:** Impact of training on self-reported confidence and correlations between knowledge test scores and confidence.

Topic area	Outcome		District A	District B
HAST	Change in aggregate confidence rating	Number of respondents	19	32
		Median (IQR)	4 (1 to 6)	3 (1 to 4)
		Wilcoxon test	z = -2.89, p = 0.004	z = -4.50, p<0.001
	Correlation post-test knowledge and post-intervention aggregate confidence rating	Number of respondents	14	16
		Tau-b	Tau-b = -0.44, p = 0.051	Tau-b = -0.14, p = 0.541
WSRHR	Change in aggregate confidence rating	Number of respondents	25	32
		Median (IQR)	3 (2 to 6)	3.5 (3 to 5)
		Wilcoxon test	z = -4.20, p<0.001	z = -4.95, p<0.001
	Correlation post-test knowledge and post-intervention aggregate confidence rating	Number of respondents	16	16
		Tau-b	Tau-b = 0.14, p = 0.531	Tau-b = 0.28, p = 0.200

Notes: The possible range of confidence ratings at each time point (i.e. before and after training) is 3 [low] to 15 [high]). Sample sizes are dependent on CHWs responding to all confidence rating questions. Numbers are different because not all respondents completed all confidence score items and because different Solomon sub-groups were eligible for different comparisons as per Table 1.

CHWs gave consistently high ratings of their experience of participating in the educational workshops. Over 95% of respondents rated their experience of the workshops, the appropriateness of their content, their interest and value to others as ‘definitely’ or ‘quite’ satisfactory. Understanding of course content was similarly rated for WSRHR, but about 85% for the HAST workshop, even though knowledge gains were similar across topics, as reported above.

The relationship between self-rated confidence and post-test scores was explored. Kendall’s tau-b correlation coefficients between aggregate confidence ratings at each time point for each workshop were explored and the results are shown in Table 4. None of the relationships are statistically significant, suggesting that CHWs may not have good insight into their own knowledge-base (self-rated confidence does not seem to be related to knowledge).

### Costs of providing training

The costs of the workshops are given in Excel S4 File, which could be used by others to estimate local costs. Excluding CHW time costs and using local currency unit exchange rates, the cost of the training was US$48 per CHW per day (or US$65 including CHW time costs).

## Discussion

Neither of two systematic reviews from the Cochrane Library concerning CHWs (sometimes known as lay health workers)–a qualitative study of barriers and facilitators [9] and a quantitative study on effectiveness [3]–cites articles dealing specifically with the effectiveness of education. However, the qualitative study states that, among many other barriers: “*[Lay health workers] described insufficient*, *poor quality*, *irrelevant and inflexible training programmes*, *calling for more training in counselling and communication and in topics outside their current role*, *including common health problems and domestic problems*.”[9] A recent review [8] found that there are many organisations who provide training for CHWs but few evaluations of CHW training programmes. Even fewer provide detail on training costs. A 2010 report from the Global Health Workforce Alliance also listed inadequate education as one of the factors that limited the success of CHW programmes.[17]

This study provides preliminary evidence that considerable improvement in CHWs’ knowledge test scores can be achieved by means of an inexpensive, and hence potentially affordable, training intervention, although the interpretations of gains must be tempered given the low internal consistency of the tests. The evidence presented shows that health literacy at pre-test (i.e. before any intervention) was generally low, but varied considerably, not only between individuals, but also between the two districts. The data suggest that (over the range observed in this study) CHWs with the lowest pre-test scores gained the most from the training. Self-confidence improved with training but, in line with the previous psychological literature,[18] individual CHWs’ self-rated confidence did not correlate with test scores. Contrary to expectations, pre-test scores on the less familiar topic group (WSRHR) were not lower than for HAST and improvements across the two topics were of a similar magnitude. In part this may be explained by the poor levels of routine training–about half of all CHWs in the North West province of South Africa had not received any training according to one study.[7] The detailed analysis of test scores also helped us identify areas where further training–or revisions to the current training prior to roll-out—would be useful (referral pathways for HAST and cervical smears for WSRHR).

The results concerning 'reactivity’ to the test were more surprising. One of the two districts showed strong apparent ‘reactivity’ while the other showed none at all. We conducted an ‘ex-post’ (retrospective) enquiry,[19] with telephone interviews with the eight participants in Solomon sub-group 3 (whose test scores had improved dramatically despite not receiving the intervention). Six of the eight CHWs disclosed that they had made notes during the test, studied the subject between tests and conferred with others. It transpired that they had got together as a group with a local NGO officer to learn more. We speculate that this happened in district B rather than in district A because residents in district B live in closer proximity and because they were not convinced they would receive the intervention after taking the second test (which they did due to our modification of the original Solomon design). Whether improvement brought about by such independent study should be called ‘reactivity’ is moot. On the one hand the extra-mural study was a reaction to the test and can be ascribed to it, an example of “assessment driving learning”. On the other hand the ‘reactivity’ was not mediated by the cognitive effect of the test *per se* and so could also be labelled as a form of ‘co-intervention’. Either way, the possibility that people may demonstrate this sort of initiative in response to testing is informative and would not have come to light but for the Solomon design.

Ignoring inflation and development costs, the training (US$48 per CHW per day) was relatively low cost, with the daily cost around two-thirds that of the didactic training element of a course to train CHWs to administer Depo-Provera in Zambia reported by Chin-Quee and colleagues,[20] and one-third of that reported for a programme concerned with hypertension in South Africa,[21] although the class size in the latter programme was just six CHWs. However, McCord and colleagues’ detailed costing study of CHWs in rural sub-Saharan Africa includes a budget of around $45 per CHW per year for training (around 1.2% of the total cost per-CHW), which suggests that only one day of our training (rather than four) could be provided by a typical CHW programme, and that this would be the only training received during a one year period. As the costs documented in our study are low, the training–if affordable—has the potential to be cost-effective if improvements in knowledge are translated into improvements in the quality of care provided by CHWs (and therefore clinical outcomes). If this is the case, it could be argued that increased investment in CHW training would be desirable, although finding the resources for such additional training is not without difficulty.[22] As suggested above, the cost per CHW will be lower with larger class sizes. The size of CHW training groups is often much larger than in our study, and this could have a negative impact on knowledge acquisition if there is less one-on-one interaction with the tutor. The relative effects on costs and outcomes would need to be considered before making decisions about optimal class size: we would expect non-linear relationships between class size and both costs and outcomes.

There are limitations to the inferences that can be made from our results. First, our observations relate to test scores only and we do not have quantitative estimates of effects on the safety, effectiveness and acceptability of care. That said, knowledge acquisition is a necessary, albeit not sufficient, condition for improved practice. Second, we measured results in the short-term only and therefore have no information on knowledge retention in the longer term. Again, a training course cannot be effective in improving knowledge in the long-term if it is ineffective in improving knowledge in the short-term: there is a need to study methods to enhance retention of knowledge (including use of information technology to deliver continuing or refresher education remotely). Third, we have not compared different methods of education–the intensity, curriculum, duration, delivery method, class size, use of technology,[23] and many other intervention design variables–that could be tested in future comparative studies. Nevertheless, the pre-test scores, variances, intracluster correlations and effect sizes observed in our study will be useful to others proposing further trials of educational interventions for CHWs. Fourth, we used the same test before and after the intervention in keeping with studies elsewhere.[8] While this ensures that both tests were of equal difficulty it limits ability to control for the ‘priming’ effect of the pre-test, whereby CHWs focus their attention during training on the areas covered in the pre-test. Future studies should consider using a post-test that includes both new and pre-test questions. Fifth, while our intervention was adapted to local circumstances and language, conforming with good practice cited elsewhere,[23] training was led by just one highly-skilled educator. Future studies should seek to draw on a larger number of trainers to evaluate likely effectiveness and not just efficacy. However, the informal training obtained by CHWs from Solomon sub-group three in district B appeared to be as effective as the study intervention, suggesting that our results are generalizable. Sixth, the intervention evaluated here focussed on a sub-set of the technical competencies CHWs need and we did not target attitudes and motivation. That said, the intervention was realistic in scope and addressed health service priorities. Seventh, CHW training is only one component of a successful CHW program, which depends also on selection of CHWs, support from community leaders, appropriate incentives, and integration with health services. Eighth, we measured knowledge, confidence and satisfaction but this excludes (at least partially) putative socio-cultural impacts of educational interventions.[24] We are mindful of this point and will later report a qualitative follow-on study of participants and their clients. Finally, our participants were selected as those first to respond to our invitation, so may have been particularly motivated to learn.

## Conclusions

In conclusion, we note that the training curriculum and delivery process were developed following careful consultation with experienced educators, health professionals and CHWs themselves. We believe these activities helped us to achieve a sharp increase in knowledge accompanied by an increase in confidence and could be considered a necessary step in acquisition of knowledge in the field. Given the developmental nature and ensuing limitations of our work, further study is of course required before a programme such as that described here should be rolled-out on a large scale.

## Supporting information

S1 File Knowledge questionnaires. List of HAST and WSRHR assessment questions for knowledge test.
(DOCX)

S2 File Confidence questionnaires. List of HAST and WSRHR rating questions to measure confidence in advising clients.
(DOCX)

S3 File Satisfaction questionnaires. List of HAST and WSRHR rating questions to measure satisfaction.
(DOCX)

S4 File CHW workshop costs. Costs of the workshops in an Excel file.
(XLSX)

## References

[1] Perry HB, Zulliger R, Rogers MM. Community health workers in low-, middle-, and high-income countries: an overview of their history, recent evolution, and current effectiveness. *Annu Rev Public Health*. 2014; 35: 399–421. 10.1146/annurev-publhealth-032013-182354 24387091

[2] Schneider H, Hlophe H, van Rensburg D. Community health workers and the response to HIV/AIDS in South Africa: tensions and prospects. *Health Policy Plan*. 2008; 23(3): 179–187. 10.1093/heapol/czn006 18388133

[3] Lewin S, Munabi-Babigumira S, Glenton C, Daniels K, Bosch-Capblanch X, van Wyk BE, et al Lay health workers in primary and community health care for maternal and child health and the management of infectious diseases. *Cochrane Database Syst Rev*. 2010(3): CD004015 10.1002/14651858.CD004015.pub3 20238326 PMC6485809

[4] One Million Community Health Workers Campaign. One Million Community Health Workers Campaign 2015. 2015. Available from: http://1millionhealthworkers.org. Accessed 17 July 2017.

[5] Thorogood M, Goudge J, Bertram M, Chirwa T, Eldridge S, Gómez-Olivé FX, et al The Nkateko health service trial to improve hypertension management in rural South Africa: study protocol for a randomised controlled trial. *Trials*. 2014; 15: 435 10.1186/1745-6215-15-435 25380994 PMC4289183

[6] Kok MC, Dieleman M, Taegtmeyer M, Broerse JE, Kane SS, Ormel H, et al Which intervention design factors influence performance of community health workers in low- and middle-income countries? A systematic review. *Health Policy Plan*. 2015; 30(9): 1207–1227. 10.1093/heapol/czu126 25500559 PMC4597042

[7] Engelbrecht JG, Letsoalo MR, Chirowodza AC. An assessment of the HIV/TB knowledge and skills of home-based carers working in the North West province in South Africa: a cross-sectional study. *BMC Health Serv Res*. 2017; 17(1): 285 10.1186/s12913-017-2238-8 28420356 PMC5395798

[8] Redick C, Dini H, Long L. The current state of CHW training programs in Sub-Saharan Africa and South Asia: what we know, what we don’t know, and what we need to do Washington, D.C: USAID; 2014.

[9] Glenton C, Colvin CJ, Carlsen B, Swartz A, Lewin S, Noyes J, et al Barriers and facilitators to the implementation of lay health worker programmes to improve access to maternal and child health: qualitative evidence synthesis. *Cochrane Database Syst Rev*. 2013(10): CD010414 10.1002/14651858.CD010414.pub2 24101553 PMC6396344

[10] Workman GM, Ribeiro RC, Rai SN, Pedrosa A, Workman DE, Pedrosa F. Pediatric cancer knowledge: assessment of knowledge of warning signs and symptoms for pediatric cancer among Brazilian community health workers. *J Cancer Educ*. 2007; 22(3): 181–185. 10.1080/08858190701428497 17760526

[11] World Health Organization. Strengthening the capacity of community health workers to deliver care for sexual, reproductive, maternal, newborn child and adolescent health Geneva, Switzerland: World Health Orgnization; 2015.

[12] McCambridge J. From question-behaviour effects in trials to the social psychology of research participation. *Psychol Health*. 2015; 30(1): 72–84. 10.1080/08870446.2014.953527 25146179

[13] Solomon RL. An extension of control group design. *Psychol Bull*. 1949; 46(2): 137–150. 18116724 10.1037/h0062958

[14] Levin H, McEwan PJ. Cost-effectiveness analysis: Methods and applications Thousand Oaks, CA: Sage; 2001.

[15] Braver MCW, Braver SL. Statistical treatment of the Solomon four-group design: A meta-analytic approach. *Psychol Bull*. 1998; 104: 150–154.

[16] World Bank Group. World Development Indicators Washington, D.C: The World Bank; 2016.

[17] Bhutta ZA, Lassi ZS, Pariyo G, Huicho L. Global Experience of Community Health Workers for Delivery of Health Related Millennium Development Goals: A Systematic Review, Country Case Studies, and Recommendations for Integration into National Health Systems Geneva, Switzerland: Global Health Workforce Alliance/World Health Organization; 2010.

[18] Kruger J, Dunning D. Unskilled and unaware of it: how difficulties in recognizing one's own incompetence lead to inflated self-assessments. *J Pers Soc Psychol*. 1999; 77(6): 1121–1134. 10626367 10.1037//0022-3514.77.6.1121

[19] Dixon-Woods M, Bosk CL, Aveling EL, Goeschel CA, Pronovost PJ. Explaining Michigan: developing an ex post theory of a quality improvement program. *Milbank Q*. 2011; 89(2): 167–205. 10.1111/j.1468-0009.2011.00625.x 21676020 PMC3142336

[20] Chin-Quee D, Bratt J, Malkin M, Nduna MM, Otterness C, Jumbe L, et al Building on safety, feasibility, and acceptability: the impact and cost of community health worker provision of injectable contraception. *Glob H Sci Prac*. 2013; 1: 3: 316–327. 10.9745/GHSP-D-13-00025 PMC4168589 25276547

[21] Gaziano TA, Bertram M, Tollman SM, Hofman KJ. Hypertension education and adherence in South Africa: a cost-effectiveness analysis of community health workers. *BMC Public Health*. 2014; 14: 240 10.1186/1471-2458-14-240 24606986 PMC3973979

[22] Taylor C, Griffiths F, Lilford R. Affordability of comprehensive CHW programmes in sub-Saharan Africa. *BMJ Glob Health*. 2017; 2(3): e000391 10.1136/bmjgh-2017-000391 29018584 PMC5623259

[23] Braun R, Catalani C, Wimbush J, Israelski D. Community health workers and mobile technology: a systematic review of the literature. *PLoS One*. 2013; 8(6): e65772 10.1371/journal.pone.0065772 23776544 PMC3680423

[24] Winters N, Oliver M, Langer L. Can mobile fhealth training meet the challenge of ‘measuring better’? *Comp Educ*. 2017; 53(1): 115–131.

