# Supplementary material for: Formative evaluation of a training intervention for community health workers in South Africa: A before and after study
Source: PLoS One. 2018 Sep 24;13(9):e0202817. doi: 10.1371/journal.pone.0202817 (PMC6152868; doi:10.1371/journal.pone.0202817)
Supplement: S1 File — List of HAST and WSRHR assessment questions for knowledge test. (DOCX) [file pone.0202817.s001.docx]

**Supporting information 1: Knowledge questionnaires**

**HAST assessment questions**

|  | **Question** |  |  |  |
| --- | --- | --- | --- | --- |
|  |  | **Yes** | **No** | **Don’t know** |
| 1 | Is Ebola an illness that is part of HAST? |  |  |  |
| 2 | Is HIV an illness that is part of HAST? |  |  |  |
| 3 | Is Malaria an illness that is part of HAST? |  |  |  |
| 4 | Are sexually transmitted infections illnesses that are part of HAST? |  |  |  |
| 5 | Is TB an illness that is part of HAST? |  |  |  |
| 6 | Can a community health worker give health promotion talks in community halls? |  |  |  |
| 7 | Can a community health worker prescribe medication? |  |  |  |
| 8 | Can a community health worker provide information to households about staying healthy? |  |  |  |
| 9 | Can a community health worker provide support for medication adherence to clients? |  |  |  |
| 10 | Can a community health worker refer their clients to other health professionals for medical services? |  |  |  |
| 11 | Does HIV cause AIDS? |  |  |  |
| 12 | Is HIV a bacterial infection? |  |  |  |
| 13 | Is HIV caused by a virus? |  |  |  |
| 14 | Is HIV most commonly transmitted through unprotected sex? |  |  |  |
| 15 | Is the medication taken to manage HIV infection called antiretroviral therapy? |  |  |  |
| 16 | Is a persistent cough a symptom of pulmonary TB? |  |  |  |
| 17 | Is a rash over the legs a symptom of pulmonary TB? |  |  |  |
| 18 | Is difficulty seeing a symptom of pulmonary TB? |  |  |  |
| 19 | Are night sweats a symptom of pulmonary TB? |  |  |  |
| 20 | Can clinics screen clients for TB? |  |  |  |
| 21 | Should community health workers refer known HIV positive community members for regular TB screening? |  |  |  |
| 22 | Is there a cure for TB? |  |  |  |
| 23 | Is TB caused by a bacterial infection? |  |  |  |
| 24 | Is TB caused by having unprotected sex? |  |  |  |
| 25 | Should a community health worker provide medication adherence support to any of their caseload who are infected with TB? |  |  |  |
| 26 | Can a client develop resistant TB if they do not take all their medication? |  |  |  |
| 27 | After taking treatment for pulmonary TB for 2 weeks if is a client no longer infectious? |  |  |  |
| 28 | Does pulmonary TB affect the lungs? |  |  |  |
| 29 | Is an HIV positive person at higher risk of contracting TB than others? |  |  |  |
| 30 | Can a community health worker refer a client to a clinic to be tested for any STI? |  |  |  |
| 31 | Should a community health worker promote safe sex in their communities? |  |  |  |
| 32 | Does having sex without a condom increase a person’s risk for contracting **any** STI? |  |  |  |
| 33 | Does taking the contraceptive pill stop a woman contracting **any** STI? |  |  |  |
| 34 | Is there medication available to treat STIs in South Africa? |  |  |  |
| 35 | Does South Africa have a high STI prevalence because a lot of people have HIV? |  |  |  |
| 36 | Does South Africa have a high STI prevalence because a lot of people have TB? |  |  |  |
| 37 | Does South Africa have a high STI prevalence because a lot of children in South Africa are born with STIs? |  |  |  |
| 38 | Does South Africa have a high STI prevalence because there is no mediation in the country to treat STIs? |  |  |  |
| 39 | Is South Africa’s STI prevalence higher than most other countries in the world? |  |  |  |
| 40 | Is chancroid an STI included in South Africa’s HAST response? |  |  |  |
| 41 | Is chlamydia an STI included in South Africa’s HAST response? |  |  |  |
| 42 | Is herpes an STI included in South Africa’s HAST response? |  |  |  |
| 43 | Is gonorrhea an STI included in South Africa’s HAST response? |  |  |  |
| 44 | Is HPV an STI included in South Africa’s HAST response? |  |  |  |
| 45 | Is syphilis an STI included in South Africa’s HAST response? |  |  |  |
| 46 | Could a community health worker refer a client for an HIV test to a doctor’s surgery? |  |  |  |
| 47 | Could a community health worker refer a client for an HIV test to a primary healthcare clinic? |  |  |  |
| 48 | Could a community health worker refer a client for an HIV test to a district hospital? |  |  |  |
| 49 | Could a community health worker refer a client for an HIV test to SASSA? |  |  |  |
| 50 | Could a community health worker refer a client for an HIV test to a mobile clinic? |  |  |  |
| 51 | Can antibiotics be used to treat TB? |  |  |  |
| 52 | Can anti-histamines be used to treat TB? |  |  |  |
| 53 | Can anti-inflammatories be used to treat TB? |  |  |  |
| 54 | Can anti-fungal be used to treat TB? |  |  |  |
| 55 | Can antivirals be used to treat TB? |  |  |  |
| 56 | Are women aged 10-24 the highest risk group in South Africa for HIV infection? |  |  |  |

**WSRHR assessment questions**

|  | **Question** |  |  |  |
| --- | --- | --- | --- | --- |
|  |  | **Yes** | **No** | **Don’t know** |
| 1 | Is testicular cancer associated with women’s sexual and reproductive health and rights? |  |  |  |
| 2 | Is cervical cancer associated with women’s sexual and reproductive health and rights? |  |  |  |
| 3 | Is breast cancer associated with women’s sexual and reproductive health and rights? |  |  |  |
| 4 | Is skin cancer associated with women’s sexual and reproductive health and rights? |  |  |  |
| 5 | Is ovarian cancer associated with women’s sexual and reproductive health and rights? |  |  |  |
| 6 | Can a community health worker provide pap screening promotion in communities? |  |  |  |
| 7 | Can a community health worker prescribe medication to treat cervical cancer? |  |  |  |
| 8 | Can a community health worker refer women for pap screening appointments? |  |  |  |
| 9 | Can a community health worker provide psychosocial support for women who are diagnosed with cervical cancer or an abnormal pap screen? |  |  |  |
| 10 | Can a community health worker administer an HPV vaccine to children? |  |  |  |
| 11 | Is conception a primary SRHR requirement of a woman at adolescent stage of the lifecourse? |  |  |  |
| 12 | Is good sexual health a primary SRHR requirement of a woman at adolescent stage of the lifecourse? |  |  |  |
| 13 | Is awareness of sexual and reproductive rights inside and outside of relationships a primary SRHR requirement of a woman at adolescent stage of the lifecourse? |  |  |  |
| 14 | Is breastfeeding a primary SRHR requirement of a woman at adolescent stage of the lifecourse? |  |  |  |
| 15 | Should women in mid-life be referred for SRHR services concerning coughing? |  |  |  |
| 16 | Should women in mid-life be referred for SRHR services concerning the pap smear? |  |  |  |
| 17 | Should women in mid-life be referred for SRHR services concerning family planning? |  |  |  |
| 18 | Should women in mid-life be referred for SRHR services concerning ante-natal care? |  |  |  |
| 19 | Do older women have contraceptive needs? |  |  |  |
| 20 | Should older women be referred for healthcare services associated with the menopause? |  |  |  |
| 21 | Are older women at risk of contracting HIV? |  |  |  |
| 22 | Are older women at highest risk of breast cancer? |  |  |  |
| 23 | Can menopausal older women be referred to clinics for medication? |  |  |  |
| 24 | Are women more vulnerable to contracting HIV than men? |  |  |  |
| 25 | Should women of all ages be referred to a clinic for a health check up that includes an HIV test? |  |  |  |
| 26 | Should a community health worker provide women of all ages with information and support concerning HIV and safe sex? |  |  |  |
| 27 | Is women’s sexual and reproductive health relevant to HIV care? |  |  |  |
| 28 | Should HIV positive pregnant women be advised about taking PMTCT during pregnancy? |  |  |  |
| 29 | Can cervical cancer be prevented? |  |  |  |
| 30 | Can cervical cancer be cured in everyone? |  |  |  |
| 31 | Can early signs of cervical cancer be detected by the pap smear? |  |  |  |
| 32 | Is cervical cancer caused by the virus HPV? |  |  |  |
| 33 | Does cervical cancer only affect women over the age of 65? |  |  |  |
| 34 | As South Africa is a patriarchal society, does this mean that women are vulnerable? |  |  |  |
| 35 | Is there a high prevalence of domestic and intimate partner violence in South Africa? |  |  |  |
| 36 | Should women be aware that they have the right to say no to anything they feel uncomfortable about? |  |  |  |
| 37 | If women were more aware of their sexual and reproductive health rights, would more women then have the confidence to report rape? |  |  |  |
| 38 | Should men have the right to abuse women? |  |  |  |
| 39 | Should a community health worker refer clients to primary healthcare clinics for HIV testing? |  |  |  |
| 40 | Should community health workers provide support for cancer sufferers in the home? |  |  |  |
| 41 | Should community health workers provide advice concerning the different types of contraception available? |  |  |  |
| 42 | Should community health workers provide advice concerning responsible sexual behaviour? |  |  |  |
| 43 | Should community health workers refer clients to clinics for ante natal support? |  |  |  |
| 44 | Could a community health worker refer a client abused by their partner to a primary healthcare clinic? |  |  |  |
| 45 | Could a community health worker refer a client abused by their partner to a police station? |  |  |  |
| 46 | Could a community health worker refer a client abused by their partner to a local NGO providing shelter for women and children? |  |  |  |
| 47 | Could a community health worker refer a client abused by their partner to a Thuthuzela care centre? |  |  |  |
| 48 | Could a community health worker refer a client abused by their partner to SASSA? |  |  |  |
| 49 | Could a community health worker refer a client for breast cancer screening to a clinic? |  |  |  |
| 50 | Could a community health worker refer a client for breast cancer screening to a private hospital? |  |  |  |
| 51 | Could a community health worker refer a client for breast cancer screening to the department of welfare? |  |  |  |
| 52 | Are adolescent girls at risk of HIV if they do not practice safe sex? |  |  |  |
| 53 | Should adolescent girls make sure to have the HPV vaccines before their sexual debut? |  |  |  |
| 54 | Can getting pregnant during adolescence increase a woman’s risk of breast cancer? |  |  |  |
| 55 | Should adolescent girls get pregnant to access child support grant payments? |  |  |  |
| 56 | Should adolescent girls who are HIV positive and pregnant be referred to a primary healthcare clinic for health support? |  |  |  |
