# Supplementary material for: Formative evaluation of a training intervention for community health workers in South Africa: A before and after study
Source: PLoS One. 2018 Sep 24;13(9):e0202817. doi: 10.1371/journal.pone.0202817 (PMC6152868; doi:10.1371/journal.pone.0202817)
Supplement: S2 File — List of HAST and WSRHR rating questions to measure confidence in advising clients. (DOCX) [file pone.0202817.s002.docx]

**Supporting information 2: Confidence questionnaires**

**HAST rating questions**

1. I feel confident in my ability to advise my clients on matters associated with HIV.
2. I feel confident in my ability to advise my clients on matters associated with TB.
3. I feel confident in my ability to advise my clients on matters associated with STDs.

**WSRHR rating questions**

1. I feel confident in my ability to advise my adolescent female clients on health topics associated with their WSRHR requirements.
2. I feel confident in my ability to advise my mid-life female clients on health topics associated with their WSRHR requirements.
3. I feel confident in my ability to advise my older female clients on health topics associated with their WSRHR requirements.
