# Supplementary material for: Formative evaluation of a training intervention for community health workers in South Africa: A before and after study
Source: PLoS One. 2018 Sep 24;13(9):e0202817. doi: 10.1371/journal.pone.0202817 (PMC6152868; doi:10.1371/journal.pone.0202817)
Supplement: S3 File — List of HAST and WSRHR rating questions to measure satisfaction. (DOCX) [file pone.0202817.s003.docx]

**Supporting information 3: satisfaction questionnaires**

**HAST rating questions**

1. I feel that the experience of participating in this workshop has been positive.
2. I feel that the workshop covered content that is appropriate to my role as a CHW.
3. I feel that the workshop was interesting.
4. I feel that I understood the majority of the workshop content.
5. I would recommend attending this workshop to my colleagues.

**WSHR rating questions**

1. I feel that the experience of participating in this workshop has been positive.
2. I feel that the workshop covered content that is appropriate to my role as a CHW.
3. I feel that the workshop was interesting.
4. I feel that I understood the majority of the workshop content.
5. I would recommend attending this workshop to my colleagues.
